# The perception threshold of the panda illusion, a particular form of 2D pulse-width-modulated halftone, correlates with visual acuity

**DOI:** 10.1038/s41598-020-69952-6

**Published:** 2020-08-04

**Authors:** Torsten Straßer, Anne Kurtenbach, Hana Langrová, Laura Kuehlewein, Eberhart Zrenner

**Affiliations:** 1 Institute for Ophthalmic Research, Centre for Ophthalmology, University of Tuebingen, Elfriede-Aulhorn-Straße 7, 72076 Tuebingen, Germany; 2 University Eye Hospital Tuebingen, Elfriede-Aulhorn-Straße 5, 72076 Tuebingen, Germany; 3 University Eye Hospital, Hradec Králové, Czech Republic; 4 Werner Reichardt Centre for Integrative Neuroscience (CIN), Otfried-Mueller-Str. 25, 72076 Tuebingen, Germany

**Keywords:** Perception, Vision disorders, Refractive errors, Visual system, Pattern vision

## Abstract

To call attention to the danger of extinction of the panda bear, the Lithuanian artist Ilja Klemencov created the artwork “They can disappear”. The illustration is composed of black-and-white zigzagged lines, which form the famous panda logo of the World Wild Fund For Nature (WWF) when seen from a distance. If one is too close to the artwork, it is difficult to spot the bear, however, if one steps back or takes off one’s glasses the panda suddenly appears. This led us to ask if the ability to see the panda is related to the visual acuity of the observer and if therefore, the panda illusion can be used to assess the spatial resolution of the eye. Here we present the results of the comparison between visual acuity determined using the Landolt C and that predicted from the panda illusion in 23 healthy volunteers with artificially reduced visual acuity. Furthermore, we demonstrate that the panda illusion is based on a 2D pulse-width modulation, explain its technical history, and provide the equations required to create the illusion. Finally, we explain why the illusion indeed can be used to predict visual acuity and discuss the neural causes of its perception with best-corrected visual acuity.

## Introduction

**Figure 1:**
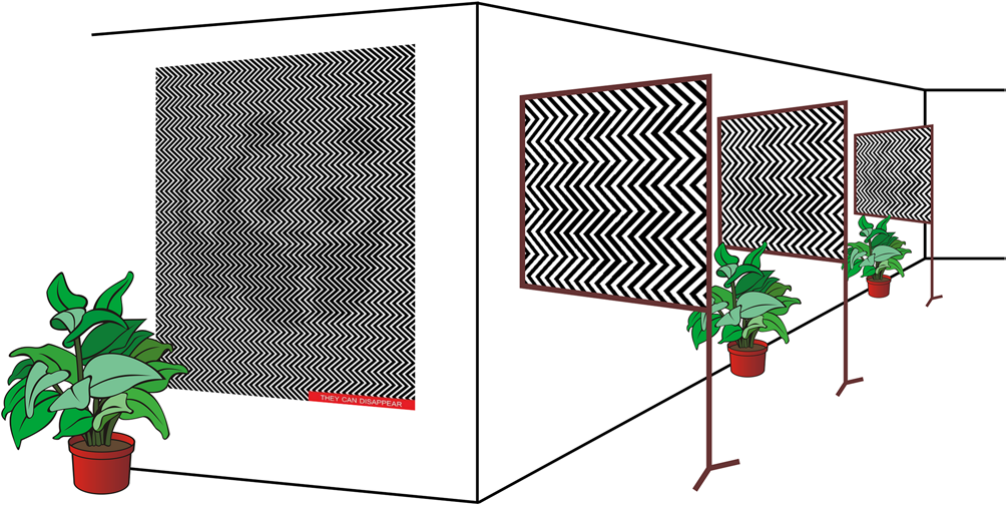
On the left, the visual illusion published by the artist Ilja Klemencov in 2016 to raise awareness about endangered species is shown. At first, the panda may not be visible, but stepping back, removing glasses, or squinting the eye will reveal the figure. The effect of the illusion becoming apparent with increasing the distance is demonstrated on the right (panda logo used with permission of the World Wide Fund For Nature).

The artwork “They can disappear”, shown in Fig. 1, was unveiled in 2016 by the artist Ilja Klemencov with the intention to raise awareness about endangered species. It features the giant panda that is used as the logo for the World-Wide Fund for Nature (WWF), hidden amongst black and white zig-zag lines. In this visual illusion, the panda may not be visible at first, but stepping back, removing glasses, or squinting the eyes will reveal the figure.

The spatial frequency of the zigzag pattern is uniform, however, in areas where the original image is black, the stripes are slightly thickened, allowing the panda to be perceived at a certain distance. Increasing the distance to the artwork leads to an increase in the spatial frequency of the zigzag pattern. Because the spatial frequency is related to visual acuity, we asked whether the panda illusion can be used as a test for visual acuity^1^.

**Figure 2:**
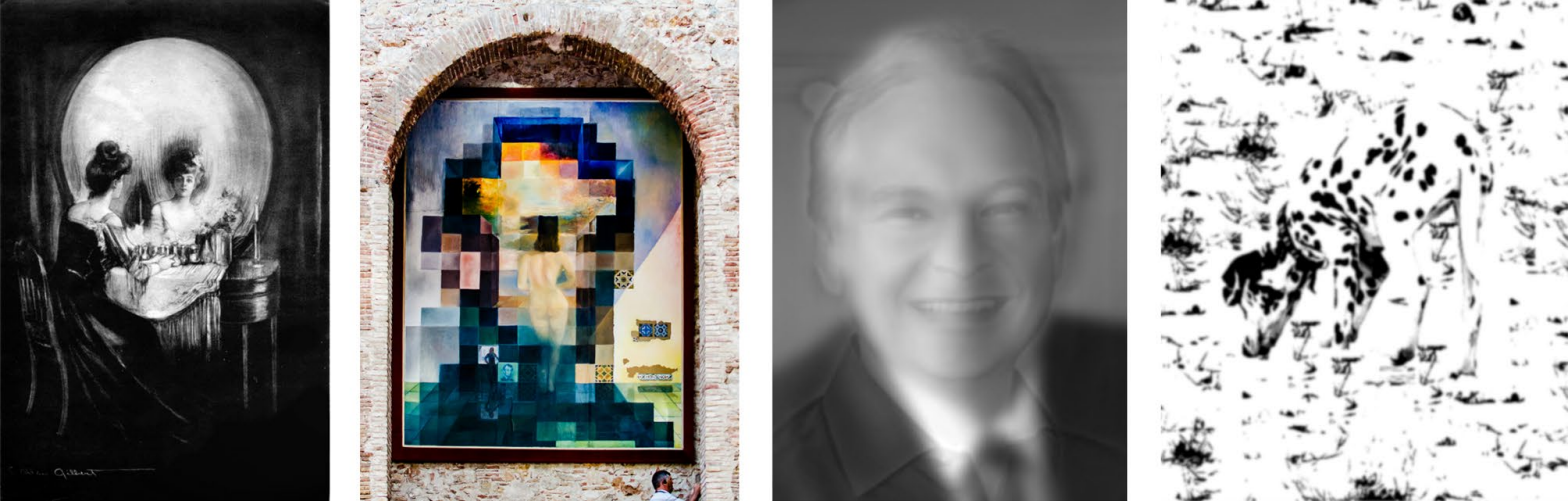
From left to right: *All is Vanity Ambiguous Figure* by Charles Allan Gilbert; photograph of the painting *Gala contemplating the Mediterranean Sea* by Salvador Dalí (Lincoln in Dalivision by Michele Ursino, CC BY-SA 2.0/cropped); a hybrid image of the first and last authors of this article; imitation of *The Dalmatian dog* illusion.

The basic working principle of the panda illusion, increasing the distance to the image or introducing blur by taking off the glasses or squinting the eyes, is similar to other artwork, like “All is Vanity Ambiguous Figure” by Charles Allan Gilbert (Fig. 2^,^ left), where the figure is perceived either as a skull or as a woman looking at a mirror. In this painting, two figures with different spatial frequencies are overlaid and a spatial low-pass filter removes the finer details of the one figure (woman), which reveals the other figure (skull)^2^. This idea was seized again recently for the creation of so-called “hybrid images”, where an image is created by merging two different images, each one spatially filtered with distinct band-pass filters, i.e. one image is filtered using a low-pass filter, the other with a high-pass filter^3–6^ (Fig. 2^,^ second from the right). By determining the distance at which the images are perceived, hybrid images can be used to assess the visual acuity of the observer^7^. A closely related illusion is used by Salvador Dalí in his painting “Gala Contemplating the Mediterranean Sea” (Fig. 2^,^ second from the left). Again, increasing the distance to the painting or blurring vision will reveal an image of Abraham Lincoln, hidden within the scene^8,9^. However, in contrast to the former illusions, in this painting the second image is conveyed by the high spatial frequencies introduced by the edges of the blocks making up the image. In this case, low-pass filtering of the image possibly increases the signal-to-noise ratio^9,10^. A comprehensive demonstration of similar illusions can be found in Nicolas Wade’s excellent book “Art and Illusionists”^11^.

Despite the fact that the panda in the panda illusion becomes visible by the same mechanisms as those in hybrid images or in Dalí’s painting, i.e. low-pass filtering by blurring or increasing the distance, it differs from them, since the figure itself is not present in the image. Instead, the image consists only of the zigzag pattern with slightly thickened lines in the black areas of the panda. In this way, the panda illusion is a more “true” visual illusion, since the thickened lines appear to create illusory contours and surfaces^12^^,^ although spatial low-pass filtering fosters the perception. In this way, the panda illusion combines features of high-frequency spatial noise, as in the Dalí painting, and those of the Dalmatian dog illusion^13,14^, a picture of a black-and-white-flecked dog on a binarized background without any noticeable boundaries (Fig. 2^,^ right). Many similar patterns producing illusory contours or surfaces are known, but any discontinuity can potentially cause their perception, as long as the distance between them is relatively small^15^. Such subjective contours often form a surface that is perceived as overlying and the illusion has been shown to be stronger for curved boundaries^16^. Nicholas Wade coined the term allusory contours for this type of illusion, which he describes as “one that hovers near the threshold of vision. It might not be seen initially, but there is always some physical discontinuity, however slight, that will define the incomplete parts of the figure alluded to”^17^. Allusory contours share in common that their content becomes apparent by some process of brightness averaging that segregates the surface of the content from the background, which is supported by blurring, e.g. by viewing the picture from afar or by defocusing the image^17^. These types of illusion are often accompanied by other illusory effects like the impression of depth and shimmering or scintillating of the underlying pattern^18^, both also present in the panda illusion.

Interestingly, the principle of these illusions has been used in the printing industry for more than 150 years, dating back to the patent granted to William Henry Fox Talbot^19^. In this process, called *halftone* printing, different shades of gray in black-and-white print media are emulated using a matrix of dots, where the diameter of the dots depends on the brightness value of the respective position in the picture to print. If the density of the dots and therefore the spatial frequency of the pattern is high enough, the visual system is not able to resolve the single dots and will interpret the image as smooth areas of a certain brightness^20^. A larger dot diameter will result in darker areas, whereas smaller diameters will cause brighter areas. The translation between the brightness level and the dot diameter is usually performed using either amplitude modulation (AM) or pulse-width modulation (PWM). An alternative approach uses a fixed dot size but modulates the density of the dots (frequency modulation, FM). The dots can be of different shapes. Common shapes are circles, squares, or diamonds, but also lines are used. Several studies have investigated the maximal dot diameter^21^, the dot profile^22^, the dot shape^23,24^, and the spatial frequency of the dot matrix^25^ with the aim to improve the visibility of halftone images and to reduce the distraction caused by the dot matrix. The panda illusion is a special case of a halftone image, in which a matrix of horizontal lines, whose lengths are modulated using PWM, are used to render the resulting image. In contrast to the common application of halftone printing, the density of the dot matrix is much lower and uses only two different shape parameters (length of the lines or duty cycles, i.e. the fraction of one period in which it is present). In addition, the phase of the matrix is modulated in relation to the position along the page-axis, resulting in the zig-zag pattern.

The aim of this study was to examine the relationship between subjective visual acuity, as measured by conventional means, and the minimum spatial frequency at which the panda illusion, extended to include several different animals, is perceived. Therefore, the subjective visual acuity of 23 volunteers was determined for both best-corrected visual acuity (BCVA), as well as artificially degraded visual acuity, and compared to the spatial frequency required to perceive the visual illusion.

## Results

### Mathematical formula for creating panda illusion-like images using pulse-width modulation

The simplest way to generate a PWM-modulated signal is to subtract the input signal from a triangular reference signal. The sign of the difference defines whether the resulting signal is in high state (on duty cycle, present) or in low state (off duty cycle, absent)^26,27^: The value of a pixel in the resulting image *I*, defined by its x/y-coordinates, is calculated as the result of the Heaviside step function *ϴ* of the difference between the carrier function, a scaled triangular function, and the source image *S* (Eq. 1), whereby the Heaviside step function is defined as in Eq. (2).

1 $$I\left( {x,y,f,\alpha ,r_{h/w} ,d_{on} ,d_{off} } \right) = \theta \left( {carrier\left( {x,y,f,\alpha ,r_{h/w} ,d_{on} ,d_{off} } \right) - S\left( {x,y} \right)} \right)$$

2 $$\theta \left( n \right) = \left\{ {\begin{array}{*{20}l} 0 \hfill & {n < 0} \hfill \\ 1 \hfill & {n \ge 0} \hfill \\ \end{array} } \right.$$

The additional parameters in Eq. (1) are used to scale the carrier function for the given duty cycles *d*_*off*_ and *d*_*on*_, which define the length of the lines of the matrix as a ratio of the period length of the triangular function for an absent or a present signal, respectively (Eq. 3).

3 $$carrier\left( {x,y,f,\alpha ,r_{h/w} ,d_{on} ,d_{off} } \right) = \frac{{triangle\left( {x,y,f,\alpha ,r_{h/w} } \right) - d_{off} }}{{d_{on} - d_{off} }}$$

From the remaining parameters *f* defines the frequency of the triangular function, whereas *α*, and *r*_*h/w*_ define the angle of the zigzag pattern between 0 (horizontal lines) and π (vertical lines), and the ratio between height and width of one tile (one zigzag element) of the zigzag pattern, respectively, and therefore the phase shift of the triangular function (Eq. 4).

4 $$triangle\left( {x, y, f,\alpha , r_{h/w} } \right) = \frac{1}{\pi }\sin^{ - 1} \left( {\sin \left( {2\pi f\left( {x - \frac{{r_{h/w} \left( {\pi - 2\sin^{ - 1} \left( {\cos \left( {\frac{2\pi fy}{{r_{h/w} }}} \right)} \right)} \right)}}{4\pi f\tan \left( \alpha \right)}} \right)} \right)} \right) + \frac{1}{2}$$

**Figure 3:**
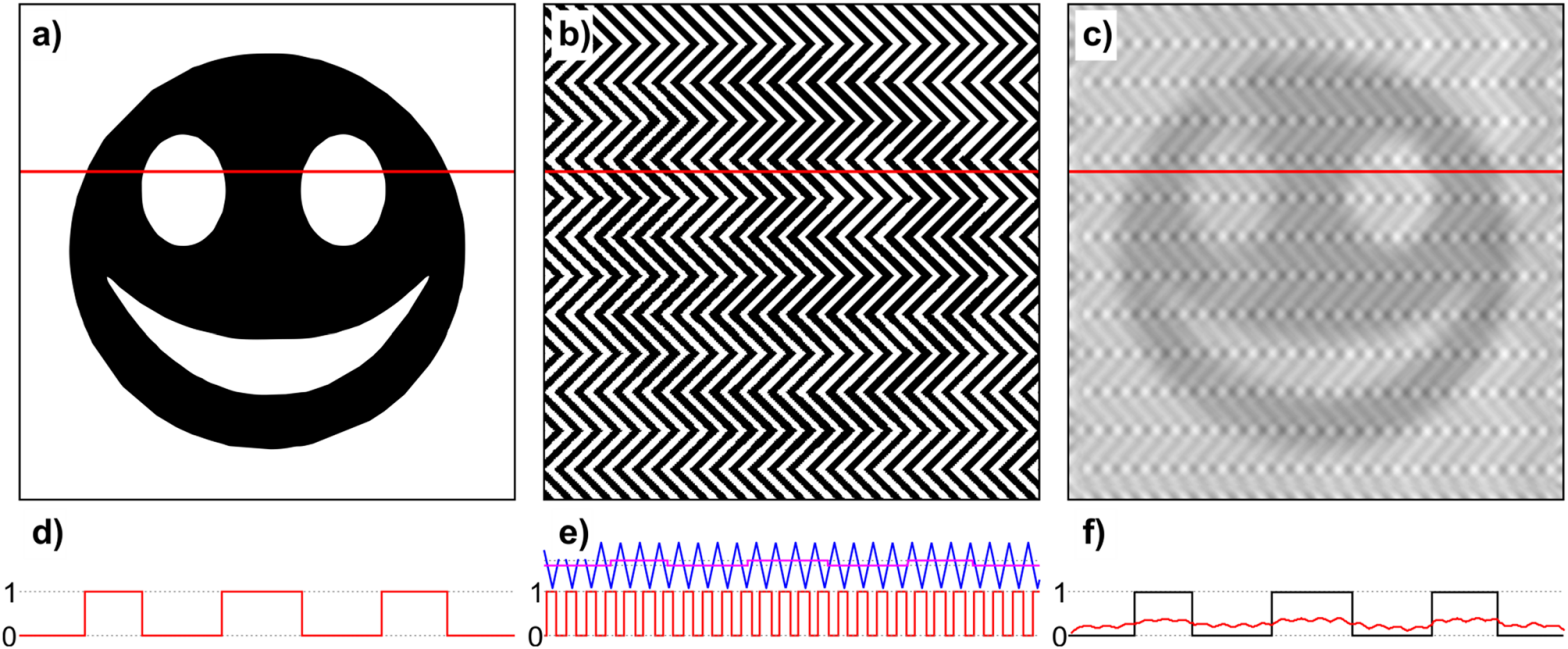
The panda illusion (**b**) is a special case of halftone printing, where the image is created using pulse-width modulation (Eq. 1 using the parameters *f* = 0.05 px^−1^ (not to be scaled), *α* = π, *d*_*on*_ = 0.6, *d*_*off*_ = 0.5, *r*_*h/w*_ = 4) from the black-and-white input image (**a**). The chart in (**d**) shows the binary luminance profile of the input image at the horizontal red line in (**a**). Similarly, the lower part in chart (**e**) depicts the pulse-width modulated binary luminance profile in the panda illusion (**b**). The upper chart in (**e**) depicts the scaled triangular carrier function (blue trace) with the input signal from the image (**a**) overlaid (pink trace). The image in (**c**) depicts the panda illusion seen with simulated degraded visual acuity of + 1 D. The chart (**f**) shows the luminance profile (red trace) of the image in (**c**), overlaid with the reconstructed signal (black trace).

Figure 3 illustrates the transformation of a binary input image (a) into the panda illusion (b) by applying Eq. (1) using the following parameters: *f* = 0.05 px^−1^ (images not to be scaled), *α* = π (90°), *d*_*on*_ = 0.6, *d*_*off*_ = 0.5, *r*_*h/w*_ = 4. The binary luminance profile (d, red trace; e, pink trace) is subtracted from the scaled triangular carrier function (e, blue trace) and results in the pulse-width modulated binary luminance profile (e, red trace). An interactive demonstration with adjustable parameters can be found in https://strator1.github.io/PandaIllusion.

Figure 3c also depicts the simulated result of an artificial degraded visual acuity of + 1 D using a Fourier-optical mathematical model^28^. Such a dioptric blur has a similar effect on the image as applying a low-pass filter^29^.

### Descriptive statistics

The results of two of the 23 subjects were excluded because of missing data, i.e. they did not complete all visual acuity or limiting spatial frequency measurements. All other subjects completed the tests successfully. Of the remaining visual acuity/limiting spatial frequency estimates, seven were classified as outliers based on their Mahalanobis distance^30,31^ (a generalized version of a distance measure in multi-dimensional space) and excluded, resulting in a total of 119 visual acuity/limiting spatial frequency pairs of 21 subjects. Table 1 summarizes the median and the quartiles of the limiting spatial frequency and the visual acuity obtained for BCVA and the different levels of artificially degraded visual acuity generated with plus lenses or occlusion foils.

**Table 1:** Median and quartiles of the limiting spatial frequency and the visual acuity of the 21 included subjects for BCVA and different levels of artificially degraded visual acuity.

Type	Level of degradation	n	Limiting spatial frequency median [Q1; Q3] (cpd)	Visual acuity median [Q1; Q3] (logMAR)
BCVA	None	18	5.1 [4.5; 5.5]	− 0.1 [− 0.2; 0.0]
Plus lens	+ 1 D	21	3.7 [3.3; 4.1]	0.4 [0.3; 0.5]
	+ 2 D	21	2.9 [2.6; 3.0]	0.7 [0.6; 0.8]
Occlusion foil	0.6	20	3.8 [3.5; 4.1]	0.1 [0.0; 0.2]
	0.2	21	3.3 [2.9; 3.5]	0.6 [0.5; 0.7]

### The effect of the limiting spatial frequency on the subjective visual acuity

Prior to utilizing the results of a linear mixed-effects model, the normal distribution of the model residuals was confirmed visually and by the Shapiro–Wilk-W test, and the homogeneity of the residual variances, the homoscedasticity^32^^,^ was ensured using the Brown–Forsythe test. The variance inflation factors (VIF) of the predictors were calculated and assured to fall below the common threshold value, indicating only acceptable collinearity between them^33^.

The fixed effects of the model (n = 119, R^2^_adjusted_ = 0.9331) explain about 61.9% of the total variability of the subjective visual acuity. The model revealed statistically significant effects of the level of degradation (*F*(4, 94.7) = 47.3023, *p* < 0.0001) and of the limiting spatial frequency on the variability of the subjective visual acuity (*F*(1, 105.9) = 4.3916, *p* = 0.0385). Neither the type of artificial degradation nor the interactions between the effects were found to have a statistically significant effect on the variability of the subjective visual acuity.

### Prediction of the subjective visual acuity from the limiting spatial frequency

**Figure 4:**
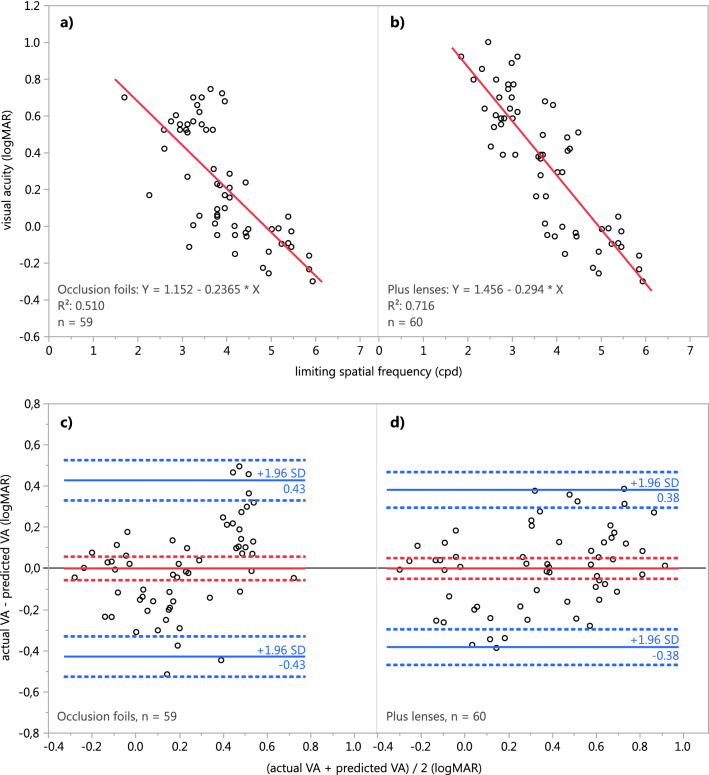
Simple linear regression of the limiting spatial frequency required to perceive the panda illusion and the visual acuity of 19 of participants with visual acuity degraded using (**a**) occlusion foils and (**b**) plus lenses and Bland–Altman plots of the predicted visual acuity based on the regression equations for (**c**) occlusion foils and (**d**) plus lenses. The solid red line depicts the mean difference, the solid blue lines the limits of agreement (LoA). Dotted lines indicate the respective 95% confidence intervals.

Simple linear regressions were calculated to predict the subjective visual acuity in logMAR based on the limiting spatial frequency for both types of artificial degradation of visual acuity. Significant regression equations were found (occlusion foils: n = 59, *F*(1, 57) = 59.2163, *p* < 0.0001; plus lenses: n = 60, *F*(1, 58) = 146.2969, *p* < 0.0001), with an R^2^ of 0.5095 for the occlusion foils and an R^2^ of 0.7161 for the plus lenses, respectively. For both types of degradation, the residuals followed a normal distribution according to visual inspection and the Shapiro–Wilk-W test and were homoscedastic^32^. Visual acuity was calculated in units of logMAR i.e. the log of the minimum angle of resolution or minimum size of a gap the subject can perceive, where 0 logMAR is equivalent to 20/20 Snellen acuity. The participants’ predicted visual acuity in logMAR is equal to 1.1519–0.2365 * (limiting spatial frequency) for degradation with occlusion foils (Fig. 4a) and 1.4563–0.2940 * (limiting spatial frequency) for degradation with plus lenses (Fig. 4b). For each increase of a cycle of a degree of the limiting spatial frequency, the participants’ subjective visual acuity increases and therefore worsens about 0.24 logMAR for occlusion foils and 0.29 logMAR for plus lenses, respectively.

The Bland–Altman analyses revealed no statistically significant difference (paired-samples t-test; occlusion foils: *t*(58) = 0.0, *p* = 1.0; plus lenses: *t*(59) = 0.0, *p* = 1.0) between the actual visual acuity and the visual acuity predicted using the equations obtained from the simple linear regressions. The mean difference and 95% confidence intervals were 0.00 [− 0.06; 0.06] logMAR for the occlusion foils (Fig. 4c) and 0.00 [− 0.05; 0.05] logMAR for the plus lenses (Fig. 4d), respectively. The limits of agreement (LoA) with 95% confidence intervals were − 0.43 [− 0.53; − 0.33] and 0.43 [0.33; 0.53] for the occlusion foils and − 0.38 [− 0.47; − 0.30] and 0.38 [0.30; 0.47] for the plus lenses.

Table 2 lists the minimum spatial frequency and the confidence interval of the zigzag pattern required for perceiving the panda illusion for commonly tested visual acuities calculated from the simple linear regressions.

**Table 2:** Minimum spatial frequency and confidence interval required for perceiving the panda illusion for common visual acuities calculated from the simple linear regressions for artificially degraded visual acuity using occlusion foils and plus lenses of 19 participants.

Visual acuity			Minimum spatial frequency [95% CI] (cpd)	
Snellen	Decimal	logMAR	Occlusion foil	Plus lens
20/200	0.1	1.0	0.57 [− 0.68; 1.31]	1.51 [1.00; 1.88]
20/100	0.2	0.7	1.85 [1.05; 2.35]	2.54 [2.21; 2.78]
20/70	0.3	0.5	2.71 [2.18; 3.05]	3.22 [3.00; 3.41]
20/50	0.4	0.4	3.14 [2.74; 3.42]	3.56 [3.37; 3.74]
20/40	0.5	0.3	3.57 [3.26; 3.82]	3.91 [3.73; 4.09]
20/30	0.6	0.2	4.00 [3.75; 4.26]	4.25 [4.06; 4.46]
20/25	0.8	0.1	4.43 [4.18; 4.75]	4.59 [4.38; 4.84]
20/20	1.0	0.0	4.86 [4.56; 5.28]	4.93 [4.70; 5.23]
20/16	1.25	− 0.1	5.29 [4.93; 5.84]	5.27 [5.00; 5.63]

## Discussion

**Figure 5:**
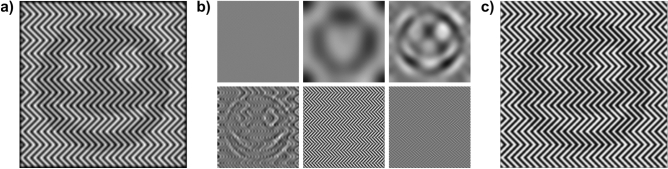
Possible mechanisms of the demodulation of the panda illusion by (**a**) summation over Ricco’s area (10 min of arc diameter), (**b**) splitting the image into six different frequency channels of about 2.8 octaves width (from to left to bottom right: 0.03–0.08, 0.08–0.22, 0.22–0.61, 0.61–1.72, 1.72–4.82, 4.82–13.49 cpd), and (**c**) multiscale filtering using the oriented difference-of-Gaussian (ODOG) model (images not to be scaled).

The results of our experiment show that blurring, induced by natural or artificial degradation of the visual acuity, up to a certain degree facilitates the decoding of the PWM-modulated visual illusion into darker and brighter areas, providing a cue for figure-ground segregation and the perception of the encoded image. However, it remains an open question, how the PWM-information is decoded in case of no external blur: with fully corrected refraction (i.e. BCVA ≥ 20/20) the illusion is perceived when the distance to it is large enough, i.e. when the spatial frequency of the zigzag pattern is higher than about 5 cpd. Based on our results, we assume that several low-pass filters along the visual pathway, either on their own or in combination, contribute to the filtering of the high-frequency content of the zigzag pattern and therefore reveal the encoded image. In this way, the Panda illusion is different from the perception of illusory contours, like Kanizsa’s triangle^34^, because the completion of the depicted figure is probably not required. This could indicate that bottom-up sensory mechanisms, such as lateral inhibition^35^, or spatial frequency analyzing mechanisms^36^ rather than cognitive top-down theories of illusory contour perception account for the panda illusion^37,38^. Kelly hypothesized the existence mechanisms for narrow-band responses of the retina related to the statistical structure of the retinal mosaic^39^. Jung and Spillmann have stressed the role of retinal receptive fields for the perceptual integration in human vision^40^ (see also Bachmann for a review^41^. Ricco’s area, the psychophysically measured extent of luminance summation underlying visual acuity, has been proposed to represent the receptive field of the retinal ganglion cells or the ganglion cell density^42–50^. Based on previous studies^45,50–52^, which determined Ricco’s area psychophysically, a range of 9 to 11 min of arc of visual angle for summation in the fovea can be assumed under photopic conditions, whereby the diameter increases in the periphery. This range corresponds to the diameter of the receptive fields (center + surround) of 5–10 min of arc in the fovea determined in humans using, amongst other tests, the Hermann grid illusion^53,54^ (see Jung and Spillmann for a review^40^ or in rhesus monkey^55^. Such a summation blurs the image similar to low-pass filtering and increases the contrast between the darker and the brighter parts of the illusion as depicted in Fig. 5a. This can be underlined by the fact that the perception of the Panda illusion is possible at lower spatial frequencies in the peripheral visual field, which corresponds to an increased Ricco’s area^49,51^ and enlarged receptive fields as well as to a lowered density of retinal ganglion cells^50^.

Low-pass filtering, and thereby removing the high-frequency content of the carrier signal, helps to reconstruct the original image from the PWM-modulated illusion (Fig. 3f). This is especially intriguing since the application of low-pass filtering in order to demodulate PWM-modulated signals is well-known in data communication^56,57^. The cut-off frequency of the low-pass filter used is directly related to the frequency of the carrier signal: a lower carrier frequency requires a lower cut-off frequency, which corresponds to the findings of our experiment, in which the results of the linear mixed-effects model show that the subjective visual acuity is related to the spatial frequency of the zigzag pattern of the panda illusion, independent of the type of the artificial degradation of visual acuity. Therefore, using simple linear regressions, it is possible to determine linear models for predicting the visual acuity from the minimum spatial frequency required to perceive the image hidden in the panda illusion: lower visual acuities require smaller spatial frequencies, higher visual acuities need a denser zigzag pattern for the detection. Once the image of the panda is perceived, it remains robust with increasing distance (an increase of spatial frequency), its perception aided by a decreasing distance between figure elements. The method is an extension of that using hybrid images to estimate visual acuity^7^ by using a quantitative measure and defined distance, instead of varying distances. Our results correspond to those of Kitakubo et al., who evaluated the distance required to perceive a circular or quadratic shape in halftone images with different dot shapes^25^. At distances larger than one meter, which corresponds to spatial frequencies larger than 8.07 cpd, the shape could be perceived reliably, whereas at the shorter distance of 0.5 m, corresponding to 4.03 cpd, the shape was not detected most of the time. This is in line with our results of a median limiting frequency of 5.1 cpd for best-corrected visual acuity (Table 1).

An additional decoding of the panda illusion may happen through spatial frequency analysis along the visual pathway, starting with different types of retinal ganglion cells, tuned to different spatial frequencies and with different spatial resolutions^58–60^, which have similarly tuned counterparts in the lateral geniculate nucleus^61,62^, though these are assumed to perform no filtering but likely act only as relays^63^. Finally, cells, tuned to narrow bands of spatial frequencies (“frequency channels”), have been found in the visual cortex^64–69^. The bandwidth of these frequency channels has been shown to be on average 2.8 octaves (the logarithm to the base of 2 of the ratio of higher to lower spatial frequencies at half-peak sensitivities^70^) in both cats and monkeys^64,66,71–73^. However, the distributions of bandwidths in a population of cells is quite broad^70^. The existence of frequency channels in the visual cortex points to a mechanism similar to Fourier analysis for the decomposition of an image into different spatial frequency components^74–76^. Such a multi-channel^77^, presumably parallel, processing of image content may provide contrast constancy, deblurring and optimize clarity of vision^78,79^. Figure 5b exemplifies the decomposition of the panda illusion into six frequency bands from 0.03 to 13.5 cpd, each with a bandwidth of 2.8 octaves. The separation into frequency channels supports the idea of a separate processing of low and high frequency content in different brain areas. It has been shown that in primates and cats single neurons in the V2/A18 and V1/A17 respond to illusory contours induced by stimuli similar to the zigzag pattern of the panda illusion, whereby the contours appear reversed in V1/A17 and depend on the relationship of the inducer spatial frequencies to the spatial filtering properties of the neurons in this areas^80^. A number of studies have demonstrated a retinotopic mapping of visual processing, with low spatial frequency content analyzed in more peripheral cortical areas^81^. Other studies found an asymmetric processing, with the right hemisphere specialized in the processing of low spatial frequencies and the left hemisphere in high spatial frequencies^82,83^. Independently, neurophysiological recordings in nonhuman primates suggest that low spatial frequency information is passed on more rapidly than high spatial frequency content^84^. This provides evidence for a coarse-to-fine approach of analyzing image content, where an initial low-pass analysis would serve to refine the subsequent processing of high spatial frequency content^85^.

The additional effects of the panda illusion at higher spatial frequencies of the zigzag pattern, a scintillating of zigzag pattern and the impression of a 3D surface, may result from the rivalry of different spatial frequency processing channels. It has been shown, that striped patterns with spatial frequency of about 3 cpd can induce perceptual illusions and distortions or even visual discomfort, whereas patterns with a frequency of about 0.3 cpd do not induce such effects^86^. These frequencies correspond to the tuning curves of V1 (optimal frequency ~ 2.8 cpd) and V2 neurons (optimal frequency ~ 0.3–0.75 cpd)^87–90^. The mismatch in the optimal spatial frequencies between the functionally connected V1 and V2 areas may result in insufficient inhibitory neural response in V2, which could manifest as a visual cortical hyper-response that results in pattern-induced illusions^88,91^.

Various models, like Laplacian of a Gaussian (LOG)^92,93^, difference-of-Gaussians (DOG)^94^^,^ Multiple Independent filters of various sizes and with both signs, half-wave Rectified before Averaging (MIRAGE)^95^, Multiple Independent Descriptions Averaged Across Scale (MIDAAS)^96^, oriented difference-of-Gaussians (ODOG)^97^ and many more, have been developed to explain the perception of visual illusions and the research is still ongoing. All of them are able to explain certain effects of some but not all visual illusions. ODOG is able to explain a wide range of visual illusion, especially ones related to perceived brightness^98^. Figure 5c depicts the output of a Python implementation of the ODOG model^99^ applied to the panda illusion. As can be seen, the ODOG model mostly acts as a low-pass filter to the panda illusion, thereby decoding the PWM-modulated signal and helping to perceive the image. However, the effect is only marginal compared to the contribution of optical blur and a possible retinal summation. The analysis of the panda illusion given here, would also apply if the illusion was represented by line luminance (pulse amplitude) modulation instead of pulse width modulation.

Shapley and Lennie^70^ provide an extensive review of the spatial frequency analysis in the visual system. Maffei and Fiorentini^100^ performed a systematic analysis of the response of neurons in the retina, the lateral geniculate, and the striate cortex to stimuli of varying spatial frequencies. For a review of the cerebral regions involved in the spatial frequency processing see Kauffmann and colleagues^85^. An overview of existing models aiming to explain visual illusions can be found by Kingdom^101^. Finally, Loffler summarizes the low and intermediate stage mechanisms of the perception of contours and shapes^102^.

The results of our experiment demonstrate, that the panda illusion works by decoding the PWM-modulated image by the visual system. Blur, resulting from the optical limitations of the eye, acts as a low-pass filter, which reveals the encoded image. The spatial frequency of the zigzag pattern at which the image is perceived, which represents the carrier frequency of the modulated information, is linearly related to the amount of blur caused by refractive error. This allows the panda illusion to be used to estimate visual acuity, and because of its counter-intuitive application—more recognized images indicate a reduced visual acuity—it can be used to cross-validate conventional acuity tests. This property renders the panda illusion especially useful in subjects with limited cooperation like young children or in subjects suspected for malingering or aggravation. However, with perfect optics, i.e. high visual acuity, the site of filtering along the visual pathway cannot be determined. In this case, low-pass filtering may occur in the retina, e.g. by summation, or it could take place in the visual cortex or higher brain areas, or, most likely, through a combination of both. An outstanding feature of the panda illusion, in contrast to similar illusions like hybrid images^4,5^, is that it consists only of one single spatial frequency. The information is encoded by modulating the duty cycle of the carrier frequency, which could render the panda illusion as a valuable stimulus to investigate further features of the visual system, like receptive field size in healthy and diseased subjects, spatial frequency channels in the cortex, or asymmetries in the processing of the image in functional studies using electroencephalography, functional magnetic resonance imaging, or functional near-infrared spectroscopy. Furthermore, the panda illusion may also be used to investigate Gestalt issues in modern neuroscience and figure-ground segregation, or employed to explore its possible origins, like perceptive field sizes, filling-in, edge polarity, or end-stopping^103^.

## Methods

### Participants

Seven male and 16 female healthy volunteers, aged between 13 and 66 (mean 38 ± 16 s.d.) years, were recruited from the staff and their relatives of the Centre for Ophthalmic Research, University of Tuebingen, Germany, after an initial basic ophthalmological examination including assessment of distant visual acuity using the Snellen acuity chart and slit-lamp examination of anterior segment of the eye. The inclusion criteria were: no ocular or systemic pathology, no abnormalities in a general ophthalmic exam and a best-corrected visual acuity (BCVA) of 1.0 (decimal) or better. Participants with a spherical error of more than ± 5 D or a cylindrical error of more than ± 3 D were not included in the study. All volunteers gave written informed consent. The study followed the tenets of the Declaration of Helsinki and was approved by the Institutional Review Board of the Medical Faculty of the University of Tuebingen (447/2016BO1).

### Stimulus presentation

**Figure 6:**
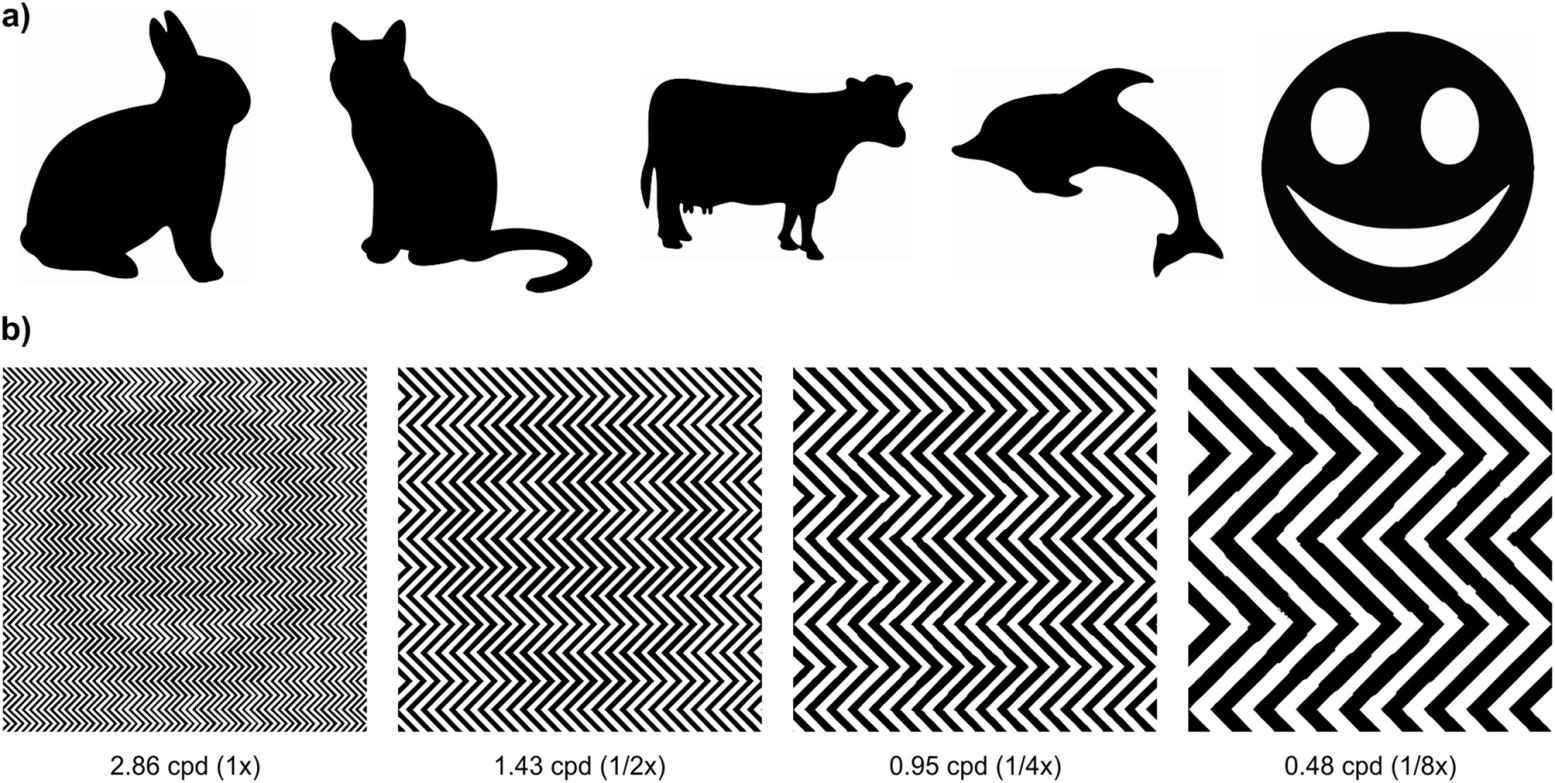
(**a**) Silhouettes (rabbit, cat, cow, dolphin, and smiley) in addition to the original panda image used as templates to generate the illusion, (**b**) examples of the generated stimuli using the smiley as a template and zigzag patterns of decreasing spatial frequency (not to be scaled). Black areas in the template image result in slightly thickened lines in the generated image.

Stimulus presentation and collection of the participants’ responses were performed using PsychoPy 1.8.3^104,105^ and a custom-developed Python 2.7 code responsible for real-time image generation. The images were created using the WWF panda logo as well as the silhouettes of four animals (rabbit, cat, cow, and dolphin), and a smiley face (Fig. 6a) by varying the spatial frequency of the zigzag pattern: black parts of the silhouette result in slightly thickened lines of the pattern (Fig. 6b). The images were presented on a high-resolution 21″ CRT monitor (1,200 × 1,600 px, Model V999, Elonex, Birmingham, UK) at a distance of 300 cm from the participant.

### Experiment

During the experiment, the participant observed the presented images with random silhouettes with one eye, while the other eye was patched. The spatial frequency of the zigzag pattern was continually decreased based on the participants’ response (two-alternative forced-choice test, 2AFC) using an adaptive staircase method^106^ until the figure became apparent. The participants were instructed to respond only if the presented figure was recognized. The maximum time to respond was 10 s. If the participant did not respond within this time, the trial was marked as missed and repeated. Between the stimulus presentations, a luminance-matched gray background was shown for 1.5 s. During the complete experiment, a red fixation cross was shown in the center of the screen. The test finished when the lowest spatial frequency, the so-called limiting spatial frequency, which allows for correct identification of the animals, was determined.

The experiment was performed with BCVA and with artificially degraded visual acuity using plus lenses (+ 1 D, + 2 D)^107^ and Bangerter occlusion foils of varying opacities (0.6, 0.2)^108,109^, which are named after a Swiss ophthalmologist who first described them.

To correlate the determined limiting spatial frequencies for the different viewing conditions, the subjective visual acuity was measured for all conditions using the Landolt-C test of the Freiburg Acuity Test (FrACT)^110^^,^ presented at the same distance on the same monitor.

### Statistical analysis

Before statistical analysis, the results of participants who had not completed all tests as well as data points classified as outliers based on their Mahalanobis distance^31^ (see “Results” above) were excluded from the dataset.

To assess the effect of the limiting spatial frequency on the variations of the subjective visual acuity measured in logMAR, a linear mixed-effect model fit by restricted maximum likelihood estimates (REML) was used (Eq. 5) utilizing the limiting spatial frequency (α), the type of artificial degradation (β) (plus lens, occlusion foil), and the level of degradation (γ) nested in type (plus lens: 0 D, + 1 D, + 2 D; occlusion foil: 1.0, 0.6, 0.2), as well as their interactions set as fixed effects and the subject (ρ) set as a random effect. For simplicity, BCVA data was replicated for both types of degradation.

5 $$VA_{logMAR} = \mu + \rho_{i} + \alpha_{j} + \beta_{k} + \gamma_{l\left( k \right)} + \left( {\alpha \beta } \right)_{jk} + \left( {\alpha \gamma } \right)_{jl\left( k \right)} + \epsilon_{ijk}$$

A simple ordinary least squares regression was used to predict the visual acuity in logMAR from the limiting spatial frequency for both artificially degraded visual acuity conditions, plus lenses and occlusion foils. Bland–Altman analyses were used to compare the visual acuity obtained from linear regression with the subjective visual acuity and to calculate the limits of agreement for the predictions^111^.

All statistical analyses were carried out using JMP 14.2.0 (SAS Institute Inc., Cary, NC, USA). Supplementary Data S1 contains raw data and analysis results.

## Supplementary information

Supplementary Data S1.

## Data Availability

All data generated or analyzed during this study are included in this published article and its Supplementary Information files (S1).
